# Response to Commentary: Diaphragmatic Atrophy After Pediatric Cardiac Surgery: Toward Integrated Monitoring

**DOI:** 10.1002/ppul.71821

**Published:** 2026-10-07

**Authors:** Clarice Laroque Sinott Lopes, Jefferson Pedro Piva

**Affiliations:** ^1^ Pediatric Intensive Care Unit Complexo Hospitalar Santa Casa de Misericórdia de Porto Alegre Porto Alegre Brazil; ^2^ Pediatric Intensive Care Unit, Hospital de Clínicas de Porto Alegre Universidade Federal do Rio Grande do Sul Porto Alegre RS Brazil

## Author Contributions

**Clarice Laroque Sinott Lopes:** writing – original draft, writing – review and editing. **Jefferson Pedro Piva:** drafting and revision.

## Funding

The authors have nothing to report.

## Conflicts of Interest

The authors declare no conflicts of interest.

To the Editor,

We thank Balkan et al. for their thoughtful reading, constructive comments, and appreciation of our work. We fully agree that the diaphragm should not be viewed merely as a passive structure undergoing disuse atrophy during mechanical ventilation. As highlighted by the authors, postoperative diaphragmatic dysfunction in pediatric cardiac surgery is multifactorial and represents a complex intersection of clinical severity, inflammatory response, ventilatory management, and sedation exposure.

Regarding the suggestion to include a comparison group with brief postoperative mechanical ventilation (e.g., early extubation or fast‐track protocols), we believe such an approach would address a distinct scientific question. Lower‐complexity patients who are rapidly extubated exhibit significantly different physiological dynamics compared to those requiring sustained mechanical ventilation. The primary objective of our study was specifically to investigate the trajectory and behavior of the diaphragm in a higher‐severity, higher‐complexity cohort—those who remain connected to mechanical ventilation for longer periods. In this vulnerable population, we found a remarkably high incidence of early‐onset diaphragmatic atrophy (56%). We consider this finding as the core of our results that need to be validated and confirmed in similar populations. Obviously, future studies are necessary to define the weight of each variable contributing to the diaphragmatic atrophy in such circumstances, as well as the preventive measures.

While we acknowledge that the extent to which ultrasound‐defined atrophy independently impacts clinical outcomes and extubation failure warrants further investigation in larger cohorts, we also agree that defining optimal cut‐off values and receiver operating characteristic (ROC) curves for functional parameters, such as the diaphragmatic thickening fraction (dTF), will be valuable steps for future research. Nonetheless, our findings reinforce that diaphragmatic ultrasonography should not be interpreted in isolation, but rather integrated as a complementary tool into multimodal bedside monitoring in these complex pediatric patients.

## Data Availability

The authors have nothing to report.

